# Factors in psychiatric admissions: before and during the COVID-19 pandemic

**DOI:** 10.1192/bjo.2021.156

**Published:** 2021-06-18

**Authors:** Robyn McCarron, Peter Swann, Fiona Thompson, Graham Murray

**Affiliations:** 1 Cambridgeshire and Peterborough NHS foundation trust; 2 University of Cambridge, Cambridgeshire and Peterborough NHS foundation trust

## Abstract

**Aims:**

The COVID-19 pandemic has impacted community mental health, but the effect on psychiatric admissions is unknown. We investigated factors contributing to acute psychiatric admissions, and whether this changed during the first UK lockdown.

**Method:**

A retrospective case-note review study with an exploratory mixed-methods design was used to examine factors in psychiatric admissions following the first UK 2020 lockdown compared to the same time periods in 2019 and 2018.

**Result:**

Themes of psychopathology, risk, social stressors, community treatment issues, and physical health concerns were generated. The mean number of codes per case was 6⋅19 (s.d. = 2⋅43), with a mean number of categories per case of 3⋅73, (s.d. = 0⋅98). Changes in routines and isolation were common factors in the study year; accommodation and substance abuse were more prominent in the control year. Relationship stressors featured strongly in both groups. There were significantly more women (χ2(1, N = 98) = 20⋅80, p < 0⋅00001) and older adults (χ2(1, N = 98) = 8⋅61, p = 0⋅0033) in the study group than the control. Single people, compared to those in a relationship (χ2(1, N = 45) = 4.46, p = 0⋅035), and people with affective disorders compared to psychotic disorders ((χ2(1, N = 28) = 5.19, p = 0⋅023), were more likely to have a COVID-19 related admission factor.

**Conclusion:**

The COVID-19 pandemic amplified pre-existing psychosocial vulnerabilities with a disproportionate psychiatric admissions impact on the mental health of women, the elderly and those with affective disorders.

